# Supplementary material for: Predicting the Occurrence of Advanced Schistosomiasis Based on FISHER Discriminant Analysis of Hematological Biomarkers
Source: Pathogens. 2022 Sep 3;11(9):1004. doi: 10.3390/pathogens11091004 (PMC9502340; doi:10.3390/pathogens11091004)
Supplement: Supplementary file 1 [file pathogens-11-01004-s001.zip › Supplementary materials File S2_Diagnostic Criteria for Schistosomiasis.pdf]

PUBLIC HEALTH IN CHINA SERIES

Series Editor Liming Li

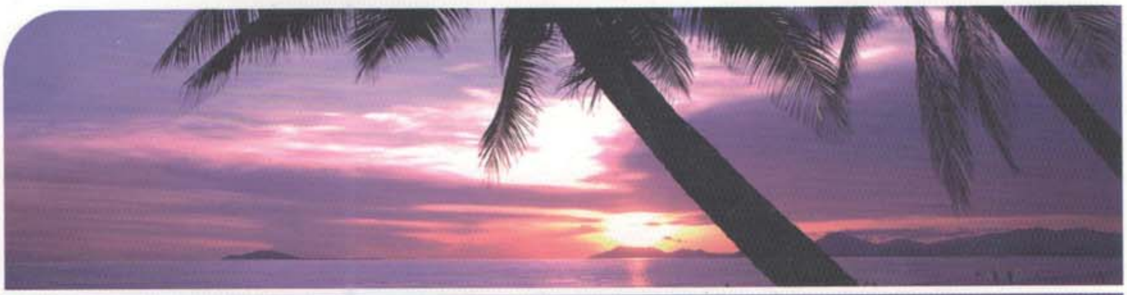

Tropical Diseases in China

Schistosomiasis

Edited by **Xiao-nong Zhou**

人民卫生出版社

PMPH PEOPLE'S MEDICAL PUBLISHING HOUSE

PUBLIC HEALTH IN CHINA SERIES

Series Editor Liming Li

Tropical Diseases in China
Schistosomiasis

Editor Xiao-nong Zhou

People's Medical Publishing House

relates with liver biopsies in pathological studies. Therefore, in addition to the clinical examination, ultrasound measurements—such as liver size, portal-vein diameter, thickness of the walls of central and peripheral portal branches, spleen size, and splenic vein diameters—improve efforts to determine the stage of the chronic schistosomiasis.

Colonoscopy examination

Adult worms live in the portal vein and its tributaries, notably the inferior mesenteric vein. Although all segments of the colon may be affected, the rectum, sigmoid and descending colon, and the domain of the inferior mesenteric vein are the main sites of pathology in more than 90% of cases. Egg deposition in the submucosa leads to granuloma formation, congestion, edema and polyp formation, and ulceration. Procto-colonoscopy examination helps to establish the diagnosis of *S. japonicum* infections, exclude similar lesions such as ulcerative and amebic colitis, and categorize the histopathological patterns.[4]

2.2.3 Diagnositic criteria for chronic schistosomiasis

Criteria

The diagnosis criteria for the clinical and confirmed chronic case of schistosomiasisi japonica has been documented as follows:

Clinical chronic case

- Lived in endemic areas or had multiple contamination of water with cercaria of schistosome
- Without symptoms, or presenting abdominal pain, diarrhea, bloody stool occasionally. Most cases have liver enlargement mainly in left lobe while a few patients present symptoms of splenomegaly
- At least one of immunological tests performed positive result

Confirmed chronic case

- Lived in endemic areas or had multiple contamination of water with cercaria of schistosome
- Without symptoms, or presenting abdominal pain, diarrhea, bloody stool occasionally. Most cases have liver enlargement mainly in left lobe while a few patients present symptoms of splenomegaly
- At least one of immunological tests performed positive result
- Found eggs or miracidium of schistosome by parasitological examination or biopsy.

DDIA

The assay is basically a chromatography technique using SEA, labeled with a dye as the indicator system. The test shows a high sensitivity and adequate specificity in healthy people. Aside from *Paragonimus westermani*, cross-reactions with other common parasitic diseases are low.[27]

Polymerase chain reaction (PCR)

Researchers have developed specific and highly sensitive PCR-based assays for the detection of *S. japonicum* DNA in feces or serum/plasma specimens of infected hosts. This potentially provides a test for early diagnosis and therapy evaluation in humans.[28] Recently, a PCR test for the detection of cell-free parasite DNA (CFPD) in human plasma has been devised. This test is showing good prospects for providing a new laboratory tool in the diagnosis of schistosomiasis in all clinical phases of the disease.[29]

2.3.4 Diagnostic criteria for advanced schistosomiasis

Criteria

Based on the Chinese National Criteria on Diagnosis of Schistosomiasis Japonica, the advanced stage of schistosomiasis japonica is divided into three types of cases: i) suspected; ii) clinically diagnosed; and iii) confirmed. For the suspected case, patients need to match the following conditions: i) have a long-term or repeated history of contact with infested water; or have a definite history of schistosomiasis treatment; and ii) clinically, have symptoms or signs regarding portal hypertension, or show indications of dwarfism or colon granuloma.

For the clinically diagnosed case, patients need supportive data from serum examination—in addition to manifesting the conditions of the suspected phase. That is, for cases without a history of schistosomiasis treatment, or with a treatment history that is more than three years ago, IHA and/or ELISA should be positive. For untreated cases or cases that have been treated at least one year ago, the serum circulating antigen should be positive.

For the confirmed case, in addition to fulfilling the conditions of the clinically diagnosed phase, patients need to manifest with eggs or miracidia detected in their feces or, in untreated cases, living eggs are found in fecal examination; in treated cases, recently modified eggs are found by rectal biopsy.[30]

Tropical Diseases in China
Schistosomiasis

Arduous efforts have been made in the last 6 decades to control and eliminate schistosomiasis in China using various control strategies, such as snail control to reduce the intensity of the disease transmission, morbidity control to reduce disease prevalence, and integrated control strategies to block the disease transmission, supported by governments at different levels.

This book analyzed the changing patterns of schistosomiasis transmission in China at different stages. Also, reviewed the scientific progress which technically support the control and surveillance of schistosomiasis in the field, summarized the working mechanism and control strategies of multi-sectoral collaboration to promote the national control programme leading to schistosomiasis elimination eventually.

人民卫生出版社
PEOPLE'S MEDICAL PUBLISHING HOUSE

世界卫生组织卫生信息和出版合作中心
WHO COLLABORATING CENTRE FOR HEALTH INFORMATION AND PUBLISHING

Acquisitions Editor: HAO Yang
Editor in Charge: LI Jiang

Cover Design: LI Xi
Format Design: LI Qiuzhai
Official website: www.pmph.com

ISBN 978-7-117-25999-6
9 787117 259996 >
