# Supplementary figures and images for: Predicting the Occurrence of Advanced Schistosomiasis Based on FISHER Discriminant Analysis of Hematological Biomarkers
Source: Pathogens. 2022 Sep 3;11(9):1004. doi: 10.3390/pathogens11091004 (PMC9502340; doi:10.3390/pathogens11091004)

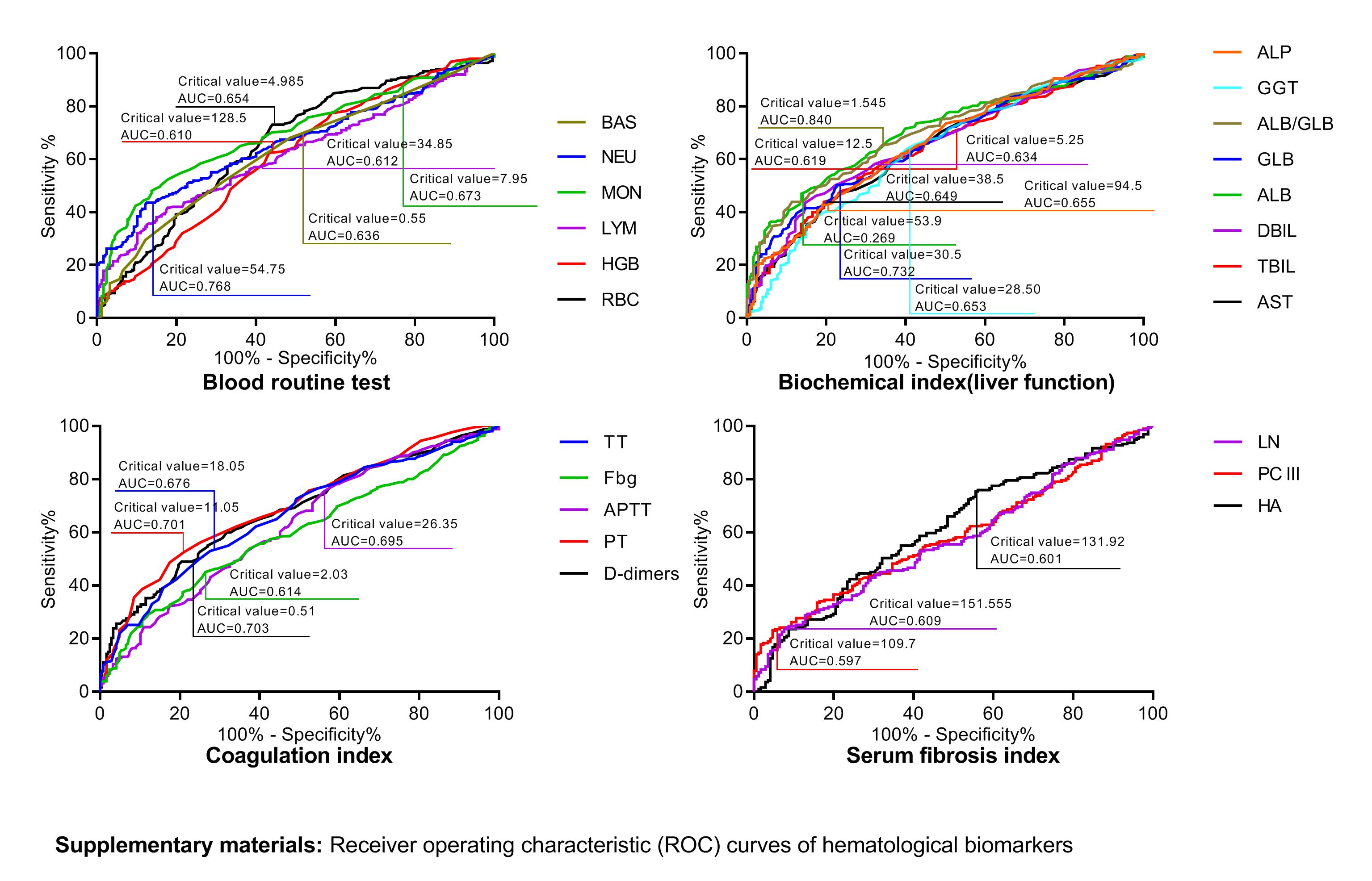

Supplement: Supplementary file 1 [file pathogens-11-01004-s001.zip › Supplementary materials File S1_ Receiver operating characteristic (ROC) curves of hematological biomarkers.jpg]
